# A Colorimetric Chemosensor Based on a Nozoe Azulene That Detects Fluoride in Aqueous/Alcoholic Media

**DOI:** 10.3389/fchem.2020.00010

**Published:** 2020-01-29

**Authors:** Lloyd C. Murfin, Kirstie Chiang, George T. Williams, Catherine L. Lyall, A. Toby A. Jenkins, Jannis Wenk, Tony D. James, Simon E. Lewis

**Affiliations:** ^1^Department of Chemistry, University of Bath, Bath, United Kingdom; ^2^School of Physical and Mathematical Sciences, Nanyang Technological University, Singapore, Singapore; ^3^Materials and Chemical Characterization (MC^*2*^), University of Bath, Bath, United Kingdom; ^4^Department of Chemical Engineering and Water Innovation & Research Centre, University of Bath, Bath, United Kingdom; ^5^Centre for Sustainable Chemical Technologies, University of Bath, Bath, United Kingdom

**Keywords:** azulene, sensor, fluoride, colorimetric, boron, water sensing

## Abstract

Colorimetry is an advantageous method for detecting fluoride in drinking water in a resource-limited context, e. g., in parts of the developing world where excess fluoride intake leads to harmful health effects. Here we report a selective colorimetric chemosensor for fluoride that employs an azulene as the reporter motif and a pinacolborane as the receptor motif. The chemosensor, NAz-6-Bpin, is prepared using the Nozoe azulene synthesis, which allows for its rapid and low-cost synthesis. The chemosensor gives a visually observable response to fluoride both in pure organic solvent and also in water/alcohol binary solvent mixtures.

**Graphical Abstract:**
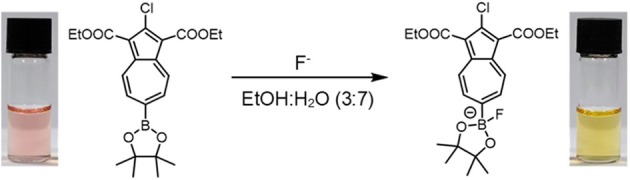
NAz-6-Bpin color change upon binding of fluoride anion.

## Introduction

Fluoride is present in naturally abundant minerals in the Earth's crust, and is therefore present in groundwater throughout the world (Ozsvath, 2009). There is only a narrow range between levels of fluoride intake that are beneficial and detrimental to human health. Whilst use of fluoridated toothpaste can help the prevention of tooth decay (Selwitz et al., 2007), exposure to greater quantities of fluoride can lead to both dental and skeletal fluorosis (DenBesten and Li, 2011; Ghosh et al., 2013). The World Health Organization (WHO) guidelines suggest that drinking water should not exceed fluoride concentrations of 1.5 mg L^−1^ (World Health Organization, 2011). For most of the world, fresh-water fluoride content is below 0.5 mg L^−1^ (World Health Organization, 1994). However, it is estimated that for over 200 million people their main source of drinking water exceeds the WHO acceptable limit of fluoride (Amini et al., 2008). Dental fluorosis is prevalent amongst the populations within areas where fluoride content of water is greater than the WHO recommended limit (Petersen et al., 2005). High ground-water fluoride content and dental fluorosis is particularly prevalent in Brazil, China, India, and throughout Africa (Fawell et al., 2006).

Whilst a range of approaches have been developed to remove fluoride from drinking water (Jagtap et al., 2012), it is necessary to have methods for detecting fluoride concentrations above the WHO safe limit, in order to know when to deploy fluoride remediation techniques. Accordingly, the development of molecular sensors for fluoride has seen significant research activity (Cametti and Rissanen, 2009; Zhou et al., 2014). Methods for chemical detection of fluoride anion may be subdivided into use of chemosensors (in which fluoride is reversibly bound to a receptor motif) and chemodosimeters (in which fluoride mediates an irreversible chemical reaction of the probe molecule). Strategies for chemodosimeter design include formation of an Si-F bond, usually inducing cleavage of an Si-O or Si-C bond (For a review, see: Chen et al., 2019. See also: Yamaguchi et al., 2000; Descalzo et al., 2002; Kim and Swager, 2003; Zhu et al., 2005; Bozdemir et al., 2010; Hu et al., 2010; Lu et al., 2011; Baker and Phillips, 2012; Li et al., 2014; Turan and Akkaya, 2014; Zou et al., 2014; Chavali et al., 2015; Mahapatra et al., 2015; Gabrielli and Mancin, 2016; Chansaenpak et al., 2018). Strategies for chemosensor design include coordination of fluoride to trivalent boron (For a review, see: Wade et al., 2010. See also: Yamaguchi et al., 2001; Sole and Gabbaï, 2004; Kim and Gabbaï, 2009; Nishimura et al., 2013; Mellerup et al., 2016; Tao et al., 2019), coordination to lanthanide complexes (Liu et al., 2014; Butler, 2015; Singhal and Jha, 2019), and fluoride-induced proton transfer and/or conformational change (Black et al., 1999; Gunnlaugsson et al., 2001, 2004, 2005; Sessler et al., 2002; Peng et al., 2005; Salman et al., 2005; Mahapatra et al., 2015).

Molecular probes for fluoride may have reporter motifs that give rise to a fluorescence, chemiluminescence, electrochemical or colorimetric response, for example. Of these, colorimetric probes for fluoride have significant advantages in the context of fluoride detection in drinking water in developing nations. They have the potential to be easy to transport and mass produce, and tests can be performed by non-expert users, without any requirement for a laboratory environment, expensive equipment or a power supply. Indeed, observing a color change does not require the user to be literate. However, such colorimetric probes are not without disadvantages. Fluoride has proven difficult to detect in water, primarily due to the extensive solvent cluster of water around fluoride (Cabarcos et al., 1999; Zhan and Dixon, 2004), with which a sensor would have to compete. In such instances, surfactants such as cetyltrimethylammonium bromide (CTAB) have sometimes been employed to dissolve the sensor in a micellular environment (Hu et al., 2010; Calderon-Ortiz et al., 2012; Elsayed et al., 2013; Roy et al., 2015; Wang et al., 2015; Qiu et al., 2016; Wallabregue et al., 2016). Phase-transfer catalysts such as tetra-*n-*butylammonium hydrogensulfate (TBAS) can be used in tandem to aid transport of the analyte into the micelle (Lopez-Alled et al., 2017).

Azulene, an isomer of naphthalene, consists of fused 7- and 5-membered ring systems. It is both unusually polar and colorful (blue) for an aromatic hydrocarbon (Michl and Thulstrup, 1976). The color of azulene can be tuned in a predictable fashion by altering the substituents at different positions on the azulene core (Liu and Asato, 2003). This fact has been exploited for its use in a range of colorimetric sensors. Examples include sensors for silver (Wakabayashi et al., 2013), nitrite (Murfin et al., 2020), mercury (Wakabayashi et al., 2007, 2008, 2012; Razus et al., 2011; Birzan et al., 2017; Buica et al., 2018, 2019), phosphate (Lichosyt et al., 2016, 2018), and reactive oxygen species (Murfin et al., 2019).

**Figure 1:**
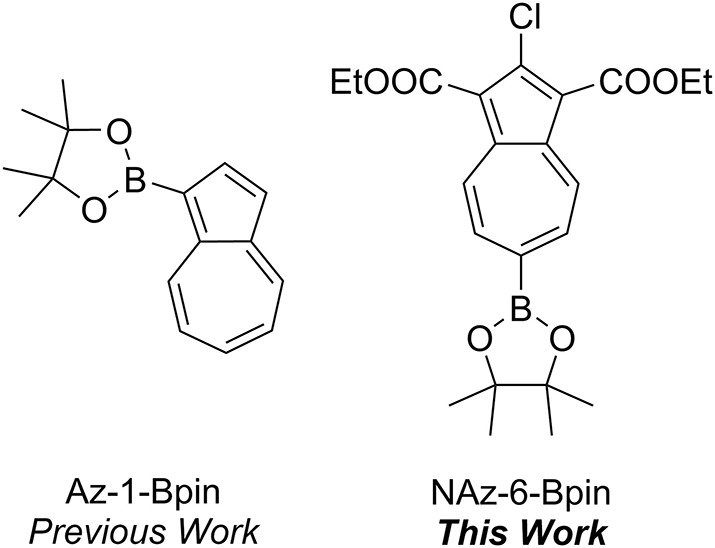
A comparison of Az-1-Bpin and NAz-6-Bpin (this work).

We have previously described the colorimetric azulene-based fluoride sensor Az-1-Bpin which exhibited excellent selectivity toward fluoride in THF (Lopez-Alled et al., 2017), with a notable color change from purple to yellow (Figure 1). The sensor did not respond to fluoride in any water/organic mixed solvent system. However, a response of Az-1-Bpin to fluoride in water was achieved in the presence of surfactants CTAB and TBAS, for which a color change from purple to purple-blue was observed. Subsequent to our work, further reports on boron-containing azulenes that respond colorimetrically to fluoride anion have been published, describing a variant of Az-1-Bpin for which a dimeric structure was claimed (Fang et al., 2018), and describing annulated borazaazulenes (Xin et al., 2020).

## Results and Discussion

The synthesis of Az-1-Bpin employs an iridium-catalyzed C-H borylation reaction (Kurotobi et al., 2003), wherein the catalyst comprises an expensive and depleting platinum group metal. Although an alternative synthesis was later reported (Bagutski et al., 2013), both syntheses require azulene itself as a starting material, which is also costly. In order to produce an azulene-based fluoride chemodosimeter that did not require azulene as a starting material, we instead considered the classical Nozoe azulene synthesis. Azulenes bearing ester substituents at the 1- and 3-positions can be readily accessed using this methodology, which employs tropolone as the starting material (Nozoe et al., 1962). We opted to employ the proven pinacolboron group as the receptor motif, but appended to the seven-membered ring of azulene (as opposed to the five-membered ring as in Az-1-Bpin). We synthesized and assessed several variants based on this design strategy, and it was found that diethyl 2-chloro-6-(4,4,5,5-tetramethyl-1,3,2-dioxaborolan-2-yl)azulene-1,3-dicarboxylate (which we have termed “NAz-6-Bpin”) gave the most substantial color change in response to fluoride.

**Figure 2:**
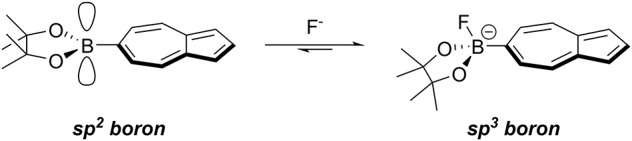
Binding of fluoride to the vacant *p*-orbital on the boronic ester results in rehybridization from sp^2^ to sp^3^.

Similarly to the case of Az-1-Bpin, our expectation was that upon fluoride binding to NAz-6-Bpin, the conjugation between the azulene ring and vacant *p*-orbital on the sp^2^-hybridized boron atom would be abolished since the boron would necessarily adopt sp^3^ hybridization. This in turn would significantly perturb the π-system and resulting in the hoped-for colorimetric response (Figure 2).

**Figure 3:**
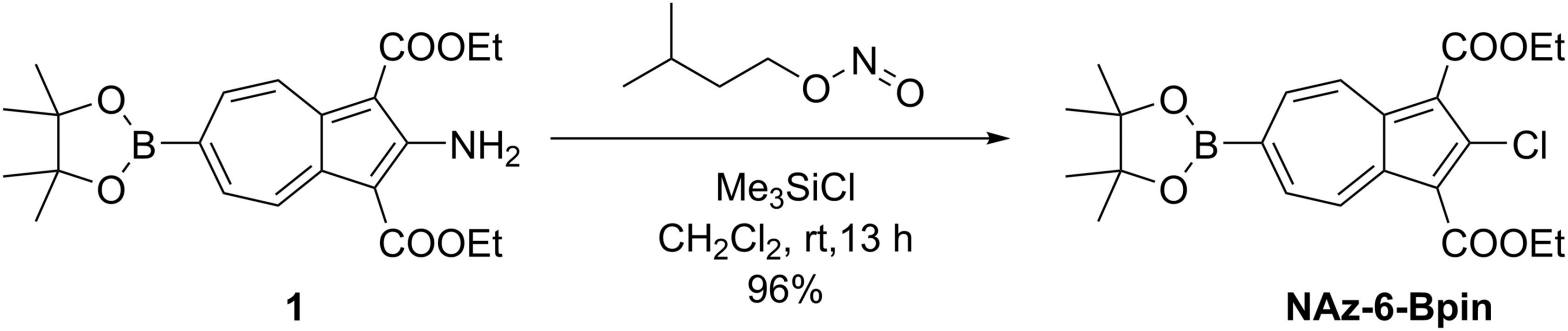
Synthesis of NAz-6-Bpin.

NAz-6-Bpin has previously been synthesized (for a purpose other than chemical sensing) through use of isoamyl nitrite and HCl gas (Xin et al., 2016). We found we could avoid the use of HCl gas by adopting a more recent procedure from the same group, using Me_3_SiCl in its place (Xin et al., 2018). Using this method, NAz-6-Bpin was synthesized from precursor **1** in a yield of 96 % (Figure 3).

**Figure 4:**
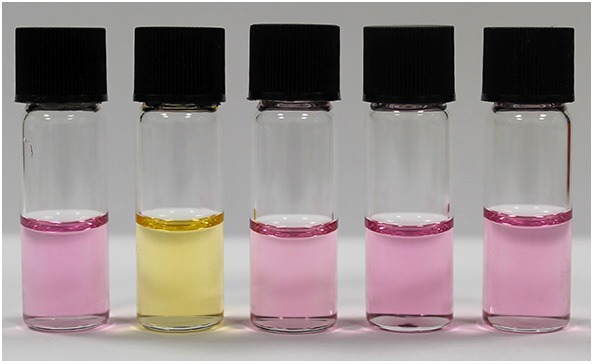
Visual selectivity test of NAz-6-Bpin in THF (0.5 mM) and halide analyte, 1:1. From left to right: no analyte, TBAF, TBACl, TBABr, TBAI. Photo taken immediately after addition of TBAX salt.

To assess the suitability of NAz-6-Bpin to detect fluoride, initial studies were performed in THF. We compared the selectivity of NAz-6-Bpin toward fluoride over other halide anions. As the experiments were performed in organic solvent, halides were added as their tetra-*n*-butyl ammonium salts (TBAX, where X = F, Cl, Br, I). An instant color change from pink to yellow was observed with TBAF only (Figure 4), and the UV-vis absorbance spectra were collected in each case (Figure S1). The absorption maximum underwent a hypsochromic shift from λ_max_ = 523 nm (NAz-6-Bpin), to λ_max_ = 464 nm (NAz-6-Bpin + F^−^). The ^11^B NMR spectra of NAz-6-Bpin and NAz-6-Bpin + TBAF in THF show two resonances, at δ = 28 and 4 ppm, respectively (Figure S2). The latter upfield shift is characteristic of a tetracoordinate boronate complex (Wrackmeyer, 2008), supporting the hypothesis presented in Figure 2.

**Figure 5:**
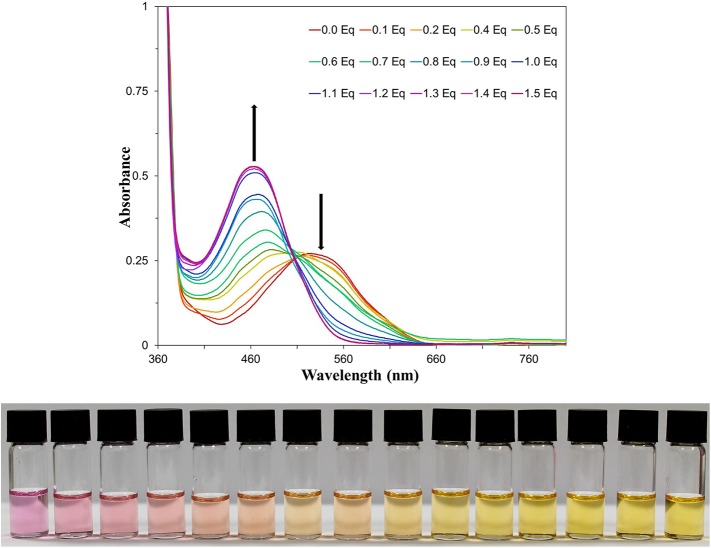
**(Top)** UV-vis titration of NAz-6-Bpin in THF (0.5 mM) with TBAF. **(Bottom)** Titration image of NAz-6-Bpin in THF (0.5 mM) with TBAF as a solution in THF. Equivalents of TBAF increase from left to right by 0.1 equivalents, starting at 0 equivalents and reaching 1.4 equivalents.

NAz-6-Bpin was then titrated against TBAF in THF (Figure 5), for which an isosbestic point at 507 nm was observed. The absorbance at 464 nm did not increase beyond the addition of 1 equivalent of TBAF, and the variation of observed absorbance at 464 nm with equivalents of TBAF shows a good linear correlation in the region from 0 to 1.0 equivalents (*r*^2^ = 0.992, Figure S3). Furthermore, a Job Plot of the interaction of NAz-6-Bpin and TBAF indicates a 1:1 stoichiometry of fluoride binding (Figure S4). With TBAF in THF, the limit of detection is 1.68 mg L^−1^ (Figure S5).

**Figure 6:**
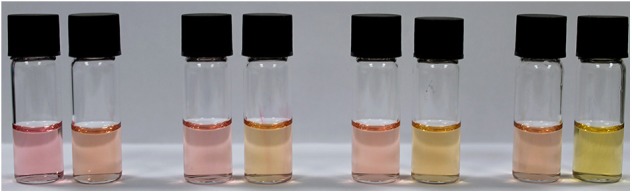
Visible assessment of color change from 1:1, v/v, aqueous/organic mixtures of NAz-6-Bpin (0.5 mM) and NaF (10 eq.). From left to right: THF, MeCN, EtOH, MeOH. Incubation time 5 min.

The ability of NAz-6-Bpin to detect fluoride in water/organic solvent mixtures was then evaluated. Exploratory experiments used a 1:1 mixture of water/organic solvent and an excess of NaF (10 equivalents), with THF, MeCN, EtOH, and MeOH being assessed (Figure 6). A mixed solvent system of DMSO/water caused NAz-6-Bpin to precipitate upon mixing. The most noticeable color change was observed in the MeOH/water system, with no further changes after 5 min. Complete UV-vis data were collected (Figure S6).

Whilst the methanol/water solvent system gave the most pronounced color change, further experiments were conducted on the EtOH/water system. It was reasoned that for the testing of fluoride by non-experts in the field, use of an ethanol-based system would prove more advantageous. Ethanol is appreciably less toxic than methanol, widely available, and low cost. The solvent ratio was explored and the optimum ratio was found to be 3:7 EtOH/H_2_O. However, the assay is robust with regard to the precise solvent ratio, with obvious color changes observed with ethanol proportions between 10 and 90% (Figure S7).

**Figure 7:**
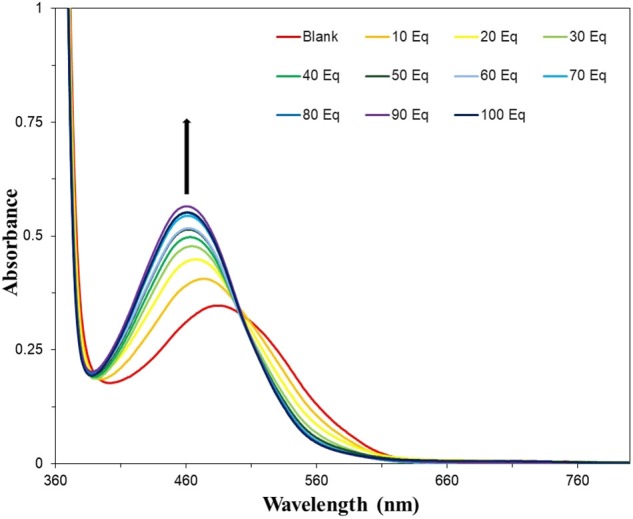
UV-vis titration of NAz-6-Bpin in EtOH/H_2_O, 3:7, v/v (0.5 mM) with NaF. Each sample was incubated for 30 min prior to acquisition of spectra.

The optimized system was then titrated with increasing concentrations of fluoride (Figure 7). An obvious color change was observed from red-pink to yellow-orange after 10–20 equivalents of NaF were added. Increased equivalents of NaF were used to determine the maximum UV-vis response (Figure S8). The requirement for an excess of fluoride to induce the maximal spectroscopic response is likely due to the extensive solvation shell of the fluoride anion in water and competition with hydroxide for binding to boron. Fitting of the data in Figure S8 to a Langmuir isotherm allowed the association constant to be determined as *K*_A_ = 214 M^−1^ at [NAz-6-Bpin] = 0.5 mM (Figure S9). The absorption maximum underwent a hypsochromic shift from λ_max_ = 486 nm (NAz-6-Bpin), to λ_max_ = 462 nm (NAz-6-Bpin + F^−^) and an isosbestic point at 502 nm was observed. NAz-6-Bpin was able to detect fluoride at neutral and basic pH levels (Figure S10). Acidic pH levels were avoided to prevent the generation of HF. Without the presence of fluoride, the sensor was stable and did not trigger a false response between pH 3–9. At pH 10, without the presence of NaF a minor color change was observed (Figure S11). NAz-6-Bpin was also assessed in a mixed ethanol: phosphate-buffered saline (PBS) solution, 3:7, v/v. It was found that the sensor changed color without the presence of fluoride, likely due to the coordination of the phosphate anion to the vacant p-orbital on the boronic ester (Figure S12). The pH stability of NAz-6-Bpin consequently means that a buffer is unnecessary. This is advantageous in the context of NAz-6-Bpin being used by non-expert users without access to such chemicals as buffer salts.

**Figure 8:**
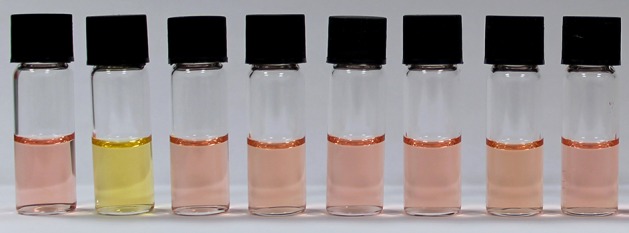
Visual selectivity test of NAz-6-Bpin in EtOH/H_2_O, 3:7, v/v (0.5 mM), with 60 equivalents of analyte used. From left to right: no analyte, NaF, NaCl, NaBr, NaI, NaNO_3_, NH_4_Cl, and Na_2_SO_4_. Each sample was incubated for 30 min prior to acquisition of spectra.

A range of anions commonly found in drinking water were also assessed in the 3:7 EtOH/water solvent system (Figure 8), for which the distinct red-pink to yellow-orange color change occurred only for NaF. A small (~6 nm) shift of the absorption maximum for Na_2_SO_4_ was observed in the UV-vis absorbance spectra (Figure S13) but this is difficult to see with the naked eye. Finally, the UV-vis limit of detection of NAz-6-Bpin and NaF in the 3:7, EtOH/water, v/v system was found to be 5.75 mg L^−1^ (Figure S14). Visually, the limit of detection was found to be 10 mg L^−1^ (Figure S15).

## Conclusion

We have designed, synthesized and evaluated an azulene-based colorimetric fluoride sensor, NAz-6-Bpin, that can successfully detect fluoride in water over other halides and common anions. The sensor has an advantage over our previously published probe Az-1-Bpin, as it is able to function in a mixed water-ethanol solvent system, without the need for any surfactant. The binary solvent system of ethanol and water used in this system renders it potentially applicable for the detection of fluoride in drinking water in the field.

## Methods

### Synthesis of NAz-6-Bpin

Under atmospheric conditions, isoamyl nitrite (0.81 mL, 6.05 mmol, 5.0 eqv) and chlorotrimethylsilane (0.78 mL, 6.05 mmol, 5.0 eqv) were dissolved in CH_2_Cl_2_ (20 mL) and left to stir for 15 min, affording a pale-yellow solution. Diethyl 2-aminoazulene-1,3-dicarboxylate **1** (0.50 g, 1.21 mmol, 1.0 eqv) was added to the solution as a single portion, causing the mixture to immediately turn dark brown and bubble rapidly, before acquiring a dark purple color over a period of 1 h. The reaction was left to stir at room temperature for 13 h, after which the volatiles were removed *in vacuo*. The crude product was purified by flushing through a silica plug, from a which a single purple band was eluted with EtOAc/Petrol (1:4) to give diethyl 2-chloro-6-(4,4,5,5-tetramethyl-1,3,2-dioxaborolan-2-yl)azulene-1,3-dicarboxylate (NAz-6-Bpin, 0.50 g, 96%) as a purple solid. δ_H_ (500 MHz, CDCl_3_) 9.54–9.42 (2H, m, H^4^, H^8^), 8.28–8.18 (2H, m, H^5^, H^7^), 4.49 (4H, q, *J* 7.1 Hz, CH_2_), 1.47 (6H, t, *J* 7.1 Hz, CH_2_CH_3_), 1.40 (12H, s, C(CH_3_)_2_). δ_C_ (125 MHz, CDCl_3_) 164.4 (C=O), 145.0 (C^2^), 142.9 (C^3a^, C^8a^), 137.04 (C^4^, C^8^), 136.99 (C^5^, C^7^), 115.3 (C^1^, C^3^), 85.3 (C(CH_3_)_2_), 60.8 (CH_2_), 25.1 (C(CH_3_)_2_), 14.6 (CH_2_CH_3_). The resonance for C^6^ was not observed. HRMS (ESI+) *m/z* calcd for (C_22_H_26_BO_6_Cl+Na)^+^, 455.1407; found 455.1437. Analytical data in agreement with those previously reported (Xin et al., 2016).

For the complete synthesis and analysis of NAz-6-Bpin, please see the corresponding Supplementary Material.

## Data Availability Statement

All datasets generated for this study are included in the article/Supplementary Material.

## Author Contributions

SL, TJ, JW, and LM conceived the project idea. LM synthesized NAz-6-Bpin. KC, LM, and GW evaluated NAz-6-Bpin as a probe for fluoride anion. LM, AJ, and KC analyzed the data. CL and LM conducted the NMR experiments. SL and LM wrote the manuscript. All authors approved the manuscript.

### Conflict of Interest

The authors declare that the research was conducted in the absence of any commercial or financial relationships that could be construed as a potential conflict of interest.
