# Supplementary material for: A Colorimetric Chemosensor Based on a Nozoe Azulene That Detects Fluoride in Aqueous/Alcoholic Media
Source: Front Chem. 2020 Jan 29;8:10. doi: 10.3389/fchem.2020.00010 (PMC7000628; doi:10.3389/fchem.2020.00010)
Supplement: Supplementary file 1 [file Table_1.docx]

A Colorimetric chemosensor based on a Nozoe azulene that detects fluoride in aqueous/alcoholic media

Supplementary Material

Lloyd C. Murfin^1^, Kirstie Chiang^1,2^, George T. Williams^1^, Catherine L. Lyall,^3^ A. Toby A. Jenkins,^1*^ Jannis Wenk,^4,5^* Tony D. James^1,5^* and Simon E. Lewis^1,5^*

^1^Department of Chemistry, University of Bath, Bath, BA2 7AY, United Kingdom

^2^School of Physical and Mathematical Sciences, Nanyang Technological University, Singapore, 637371

^3^Materials and Chemical Characterization (MC^2^), University of Bath, Bath, BA2 7AY, United Kingdom

^4^Department of Chemical Engineering and Water Innovation & Research Centre, University of Bath, Bath, BA2 7AY, United Kingdom

^5^Centre for Sustainable Chemical Technologies, University of Bath, Bath, BA2 7AY, United Kingdom

**Reagents, General Procedure and Equipment**

Solvents and reagents used were reagent grade, purchased from Fisher Scientific, Sigma-Aldrich and Fluorochem. All chemicals and solvents purchased were used without further purification. Unless stated otherwise, ambient conditions were used for each reaction. Inert conditions were achieved using anhydrous solvents and by allowing the reaction to proceed under an atmosphere of nitrogen. Anhydrous solvents were dried using an Innovative Technology PS-400-7 Solvent Purification System. Mass spectra were recorded on a microTOF mass spectrometer, with electrospray ionisation (ESI) used as the ionisation method. All ^1^H and ^13^C NMR spectra were obtained from a 500 MHz Agilent ProPulse, for which proton decoupling was active for ^13^C NMR. All chemical shift values (δ) are reported in ppm relative to tetramethylsilane (internal standard), referenced to the residual solvent peak of the deuterated solvent used. Multiplicity of the recorded peaks are listed as s for singlet, d for doublet, t for triplet, q for quartet, or m for multiplet. Coupling constants (*J* / Hz) are given where calculable. All UV/Vis experiments were performed on a Shimadzu UV-1800 UV spectrometer, for which a quartz cuvette of 1 cm path length was used. A phosphate-buffered saline (PBS) solution was generated by dissolved one “Fisher Scientific PBS Tablet” in 200 mL water to generate a solution containing 0.01 M phosphate buffer, 0.0027 M KCl and 0.137 M NaCl.

# Synthesis

## Overall Synthetic Scheme

## 7-Oxocyclohepta-1,3,5-trien-1-yl 4-methylbenzenesulfonate (**2**)

Under an atmosphere of nitrogen, tropolone **2** (5.00 g, 40.94 mmol, 1.0 eqv) and tosyl chloride (7.81 g, 40.94 mmol, 1.0 eqv) were dissolved in anhydrous CH_2_Cl_2_ (60 mL), into which NEt_3_ (5.71 mL, 40.94 mmol, 1.0 eqv) was added dropwise. The solution was further diluted in CH_2_Cl_2­_ (60 mL) to prevent a suspension from forming, and left to stir for 38 hours, forming a yellow slurry. The reaction was quenched with ice, extracted with CH_2_Cl_2_ (3 $\times$ 100 mL), dried with MgSO_4_, filtered and concentrated under reduced pressure affording 7-Oxocyclohepta-1,3,5-trien-1-yl 4-methylbenzenesulfonate **3** as a crystalline yellow solid (11.30 g, 99 %).

δ_H_ (500 MHz, CDCl_3_) 7.91 (2H, d,  *J* 8.3 Hz, H^6^), 7.46 (1H, d, *J* 9.4 Hz, H^1^), 7.33 (2H, d, *J* 8.1 Hz, H^7^), 7.21, (1H, ddd, *J* 12.3, 7.9, 1.1 Hz, H^4^), 7.15 (1H, d, *J* 12.3 Hz, H^5^), 7.08 (1H, ddd, *J* 11.6, 7.9, 1.1 Hz, H^3^), 6.98 (1H, ddt, *J* 10.9, 9.4, 1.3 Hz, H^2^), 2.45 (3H, s, CH_3_). δ_C_ (125 MHz, CDCl_3_) 179.5 (C=O), 155.3 (O-C), 145.6 (SC), 141.3 (C^5^), 136.4 (C^4^), 134.8 (C^3^), 133.6 (CH_3_C), 130.9 (C^2^), 130.0 (C^1^), 129.7 (C^7^), 128.7 (C^6^), 21.9 (CH_3_). Analytical data in agreement with those previously reported (Nolting *et al.,* 2009).

## Diethyl 2-aminoazulene-1,3-dicarboxylate (**4**)

7-Oxocyclohepta-1,3,5-trien-1-yl 4-methylbenzenesulfonate **3** (4.50 g, 16.29 mmol, 1.0 eqv) and ethyl cyanoacetate (3.82 mL, 35.83 mmol, 2.2 eqv) were dissolved in ethanol (80 mL) and cooled to 0 °C, into which *t*-butylamine (4.28 mL, 40.72 mmol, 2.5 eqv) was added dropwise. The solution was left to stir for 19 hours, during which the system warmed to room temperature and the product, an orange precipitate, had formed. The precipitate was filtered and the filtrate concentrated to ~30 mL under reduced pressure, cooled to 0 °C, into which water (100 mL) was added to further precipitate the product. The precipitate was filtered, washed with water and dried under vacuum to afford diethyl 2-aminoazulene-1,3-dicarboxylate **4** (3.80 g, 81%) as a bright orange powder (Nozoe *et al.,* 1971).

δ_H_ (500 MHz, CDCl_3_) 9.16 (2H, d, *J* 10.2 Hz, H^4^, H^8^), 7.79 (2H, br. s, NH_2_), 7.55 (2H, t, *J* 10.3 Hz, H^5^, H^7^), 7.47 – 7.41 (1H, m, H^6^), 4.47 (4H, q, *J* 7.1 Hz, CH_2_), 1.49 (6H, t, *J* 7.1 Hz, CH_3_). δ_C_ (125 MHz, CDCl_3_) 166.7 (C=O), 162.6 (C^2^), 146.3 (C^3a^, C^8a^), 133.0 (C^6^), 132.7 (C^5^, C^7^), 131.6 (C^4^, C^8^), 99.9 (C^1^, C^3^), 60.0 (CH_2_), 14.8 (CH_3_). HRMS (ESI+) *m/z* calcd for (C_16_H_17_NO_4_+Na)^+^, 310.1050; found 310.1063. Analytical data in agreement with those previously reported (Yang *et al.,* 2017).

## Diethyl 2-amino-6-bromoazulene-1,3-dicarboxylate (**5**)

Diethyl 2-aminoazulene-1,3-dicarboxylate **4** (1.00 g, 3.48 mmol, 1.0 eqv) was dissolved in anhydrous CH_2_Cl_2_ (20 mL) and cooled to 0 °C, into which bromine (0.20 mL, 3.83 mmol, 1.1 eqv) was added over a 20 min period. The solution was warmed to room temperature and stirred for 1.5 hours. The reaction was quenched with water (150 mL), separated and the aqueous layer was extracted with CH_2_Cl_2_ (2 $\times$ 50 mL). The collected organic extracts were dried with MgSO_4_, filtered and concentrated under reduced pressure. The crude product was recrystallized from toluene/ hexane to give diethyl 2-amino-6-bromoazulene-1,3-dicarboxylate **5** as a dark brown crystalline solid (1.14 g, 89%).

δ_H_ (500 MHz, CDCl_3_) 8.86 (2H, d, *J* 11.1 Hz, H^4^, H^8^), 7.82 (4H, m, H^5^, H^7^, NH_2_), 4.46 (4H, q, *J* 7.1 Hz, CH_2_), 1.47 (6H, t, *J* 7.1 Hz, CH_3_). δ_C_ (125 MHz, CDCl_3_) 166.4 (C=O), 162.5 (C^2^), 144.5 (C^3a^, C^8a^), 135.5 (C^5^, C^7^), 129.7 (C^4^, C^8^), 128.6 (CBr), 101.2 (C^1^, C^3^), 60.3 (CH_2_), 14.8 (CH_3_). HRMS (ESI+) *m/z* calcd for (C_16_H_16_BrNO_4_+Na)^+^, 388.0155; found 388.0149. Analytical data in agreement with those previously reported (Holovics *et al.,* 2006).

## Diethyl 2-amino-6-(4,4,5,5-tetramethyl-1,3,2-dioxaborolan-2-yl)azulene-1,3-dicarboxylate (**1**)

# Under an atmosphere of nitrogen, diethyl 2-amino-6-bromoazulene-1,3-dicarboxylate 5 (6.50 g, 17.75 mmol, 1.00 eqv), [1,1′-bis(diphenylphosphino)ferrocene]dichloropalladium(II) (0.65 g, 0.89 mmol, 0.05 eqv), bis(pinacolato)diboron (4.96 g, 19.53 mmol, 1.10 eqv) and potassium acetate (5.23 g, 52.25 mmol, 3.00 eqv) were combined, into which anhydrous DMSO (60 mL) was added. The mixture was heated at 80 °C for 18 hours, after which it was allowed to cool to room temperature. The mixture was diluted with water (100 mL) then extracted with CH_2_Cl_2_ (4 $\boldsymbol{\times}$ 50 mL). The combined organic extract was washed with water (200 mL), dried over MgSO_4_, filtered and concentrated under reduced pressure. The crude product was purified *via* column chromatography, eluting with EtOAc:Petrol (2:8, R_f_=0.60) to give 2-amino-6-(4,4,5,5-tetramethyl-1,3,2-dioxaborolan-2-yl)azulene-1,3-dicarboxylate (4.82 g, 66%) 1 as a bright orange powder.

δ_H_ (500 MHz, CDCl_3_) 9.06 (2H, d, *J* 11.0 Hz, H^4^, H^8^), 8.03 (2H, d, *J* H^5^, H^7^), 4.44 (4H, q, *J* 7.1 Hz, CH_2_), 1.46 (6H, t, *J* 7.1 Hz, CH_3_). δ_C_ (125 MHz, CDCl_3_) δ_C_ (125 MHz, CDCl_3_) 166.7 (C=O), 163.6 (C^2^), 147.5 (C^3a^, C^8a^), 138.7 (C^4^, C^8^), 130.4 (C^5^, C^7^), 99.8 (C^6^), 84.7 (C(CH_3_)_2_), 59.9 (CH_2_), 25.0 (C(CH­_3_)_2_), 14.7 (CH_2_CH_3_). HRMS (ESI+) *m/z* calcd for (C_22_H_28_BNO_6_+Na)^+^, 436.1902; found 436.2021. Analytical data in agreement with those previously reported (Kurotobi *et al.,* 2002).

## UV-vis Results

## **Experiments in THF**


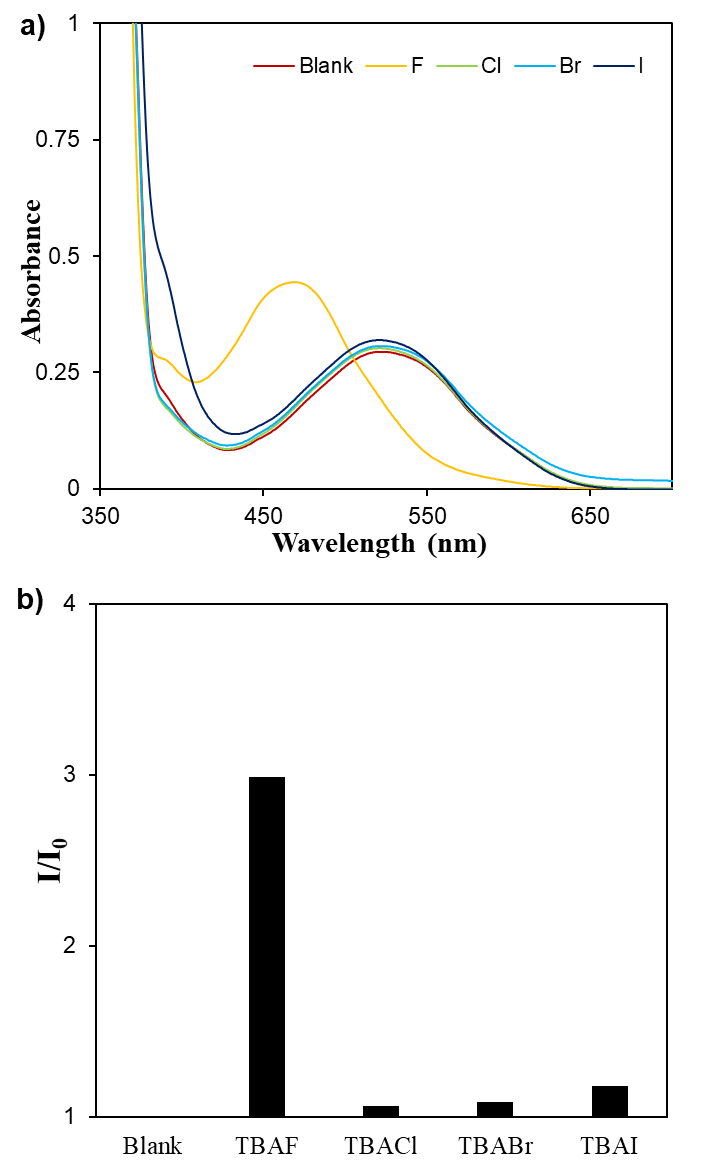

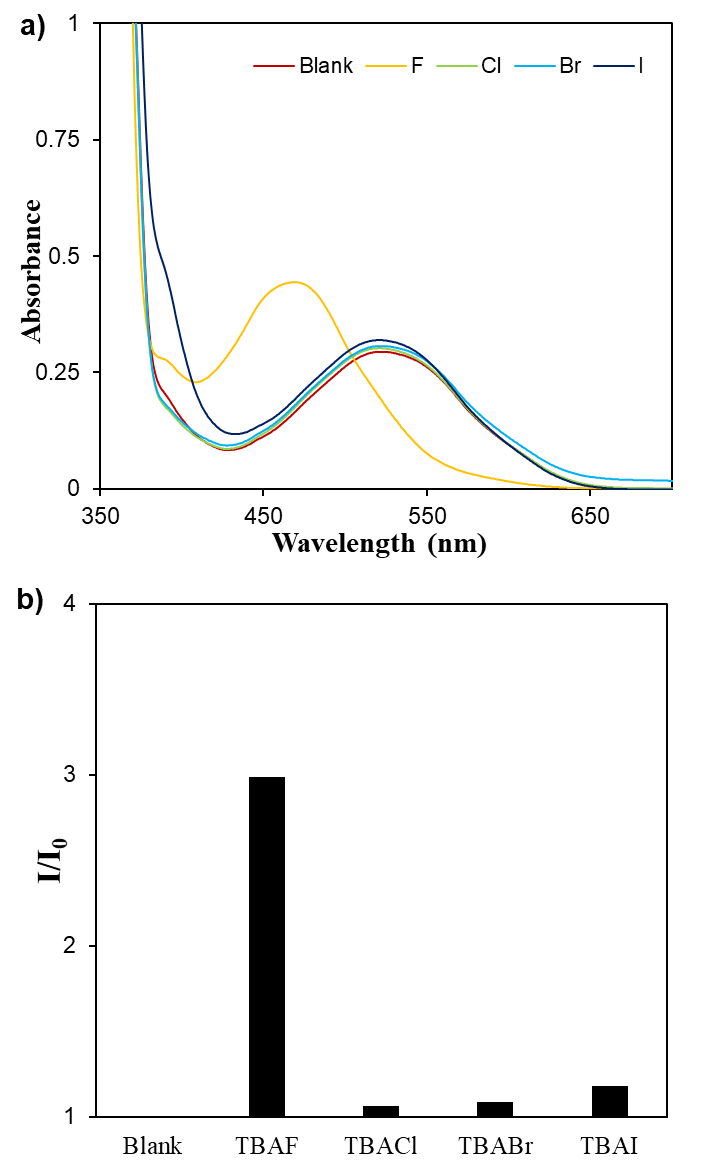


**Figure S1**. a) UV-vis selectivity test of NAz-6-Bpin in THF (0.5 mM) with TBAX (where X = F, Cl, Br, and I) at a ratio of 1:1. b) Comparison of absorbance intensity of NAz-6-Bpin and analytes at 464 nm.


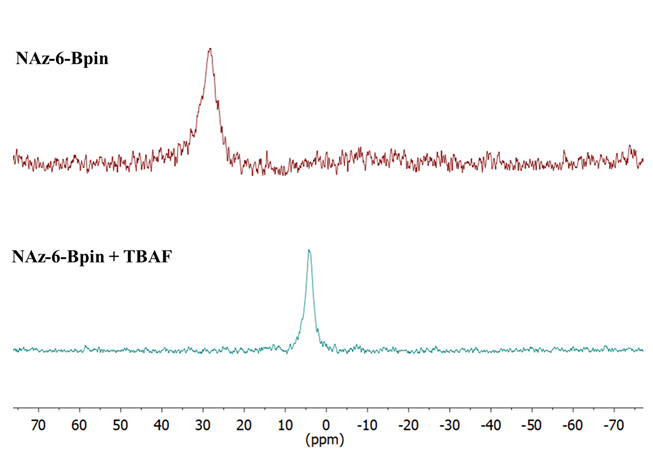


**Figure S2**. 11B NMR of NAz-6-Bpin and NAz-6-Bpin + TBAF (1:1) in THF.


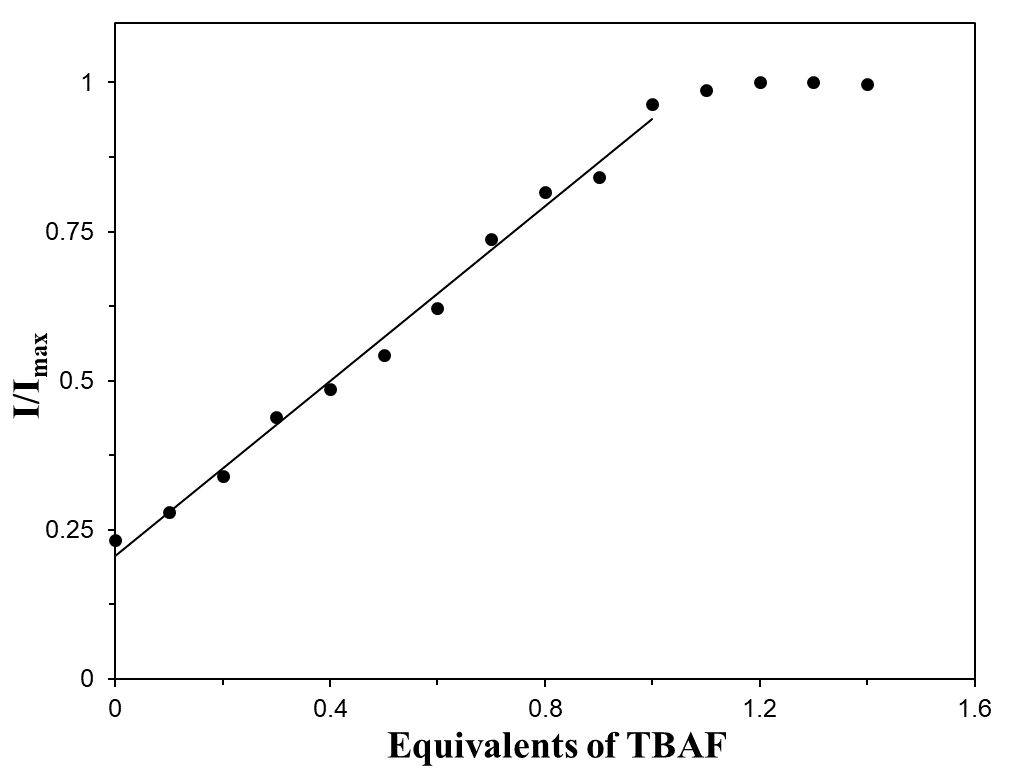


**Figure S3**. UV-vis absorbance dose dependence curve (λ_max_ = 464 nm) of NAz-6-Bpin (0.5 mM) and TBAF.


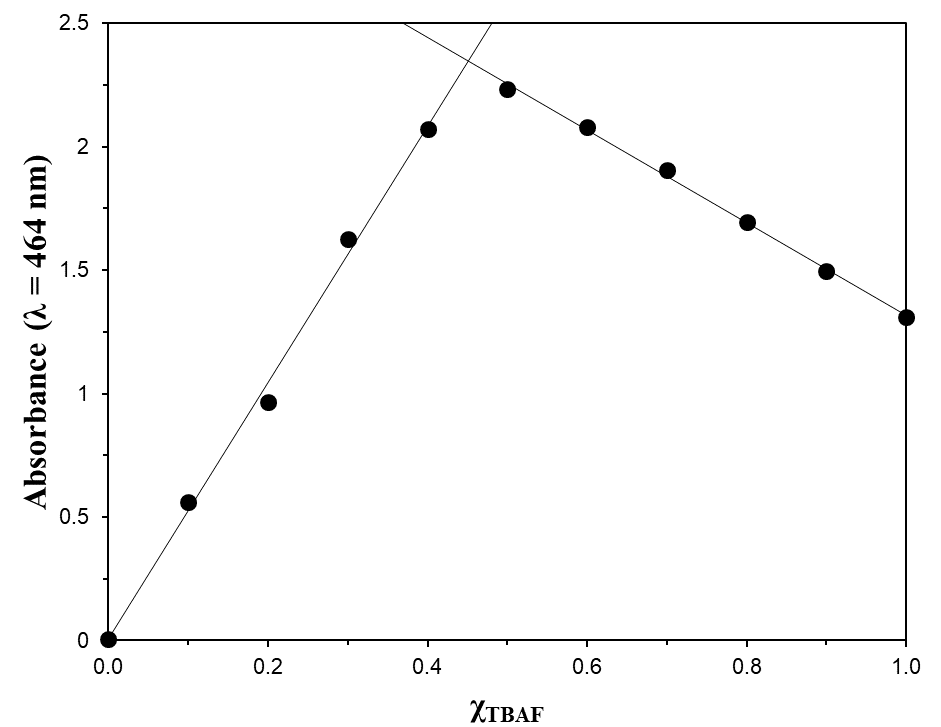


**Figure S4**. Job Plot analysis using the UV-vis absorbance at λ = 464 nm and mole fractions of TBAF.


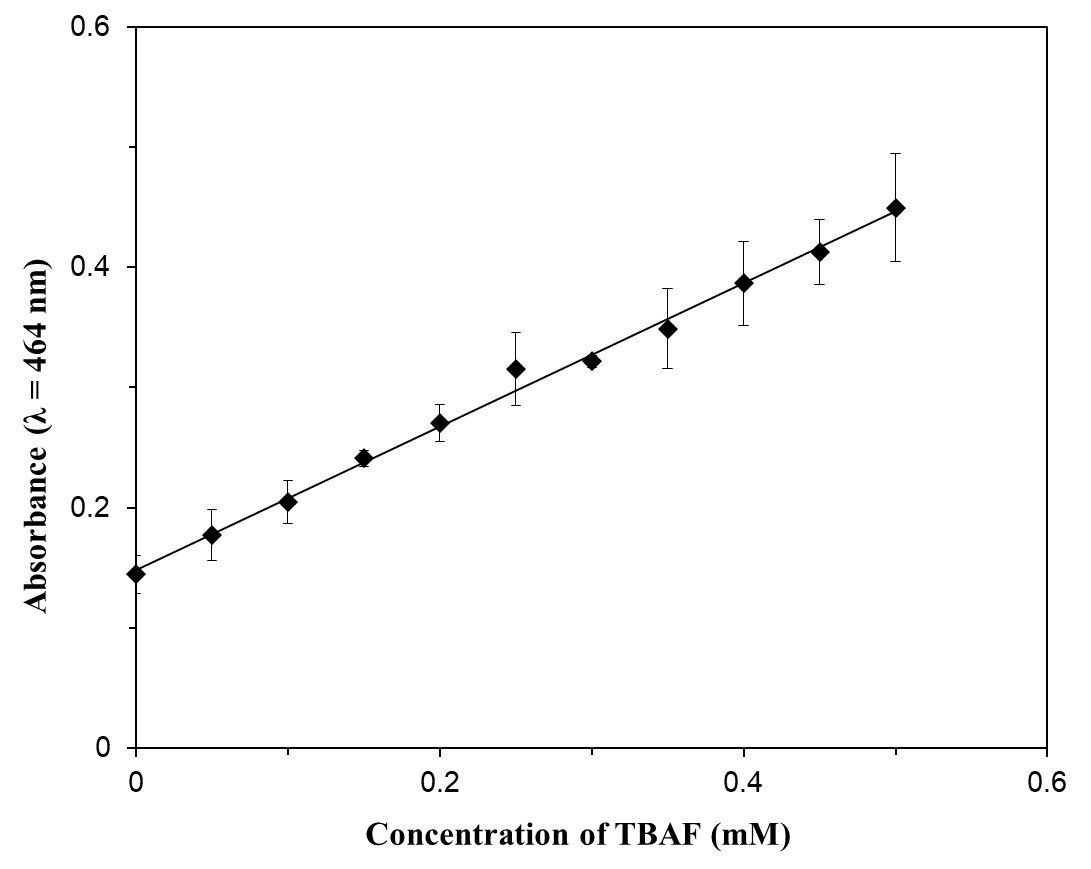


**Figure S5**. Limit of detection of NAz-6-Bpin in THF (0.5 mM) determined by UV-vis absorption at 464 nm with TBAF, calculated to be 1.68 mg L^-1^. Displayed errors calculated by standard deviation, n =3.

## **Experiments in Water Mixtures**


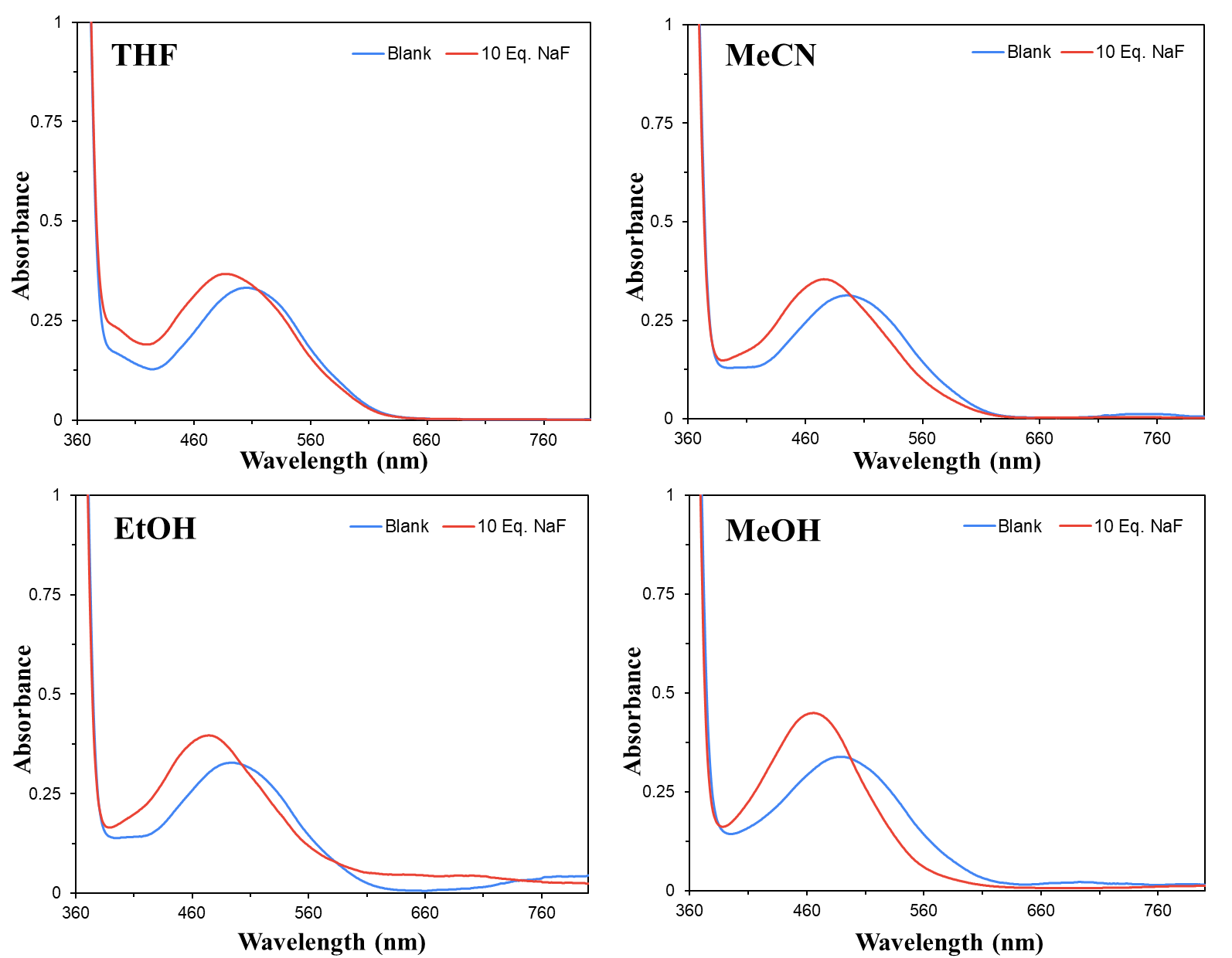


**Figure S6**. UV-vis colour change in a 1:1, v/v, aqueous:organic mixtures of NAz-6-Bpin (0.5 mM) and NaF (10 eq.).


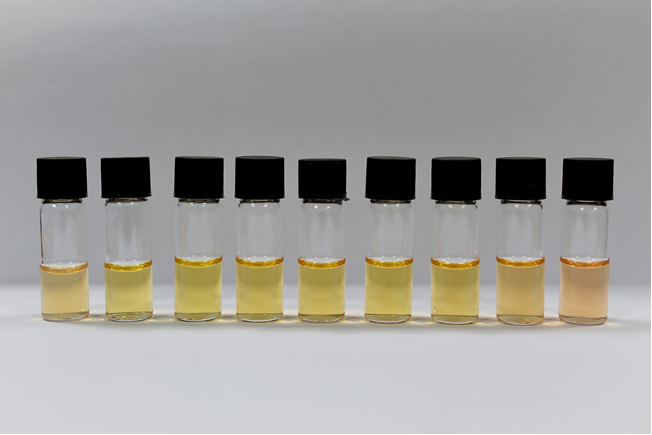


**Figure S7**. Assaying the EtOH:water ratio used for NAz-6-Bpin 0.5 mM) to detect NaF (60 eqv). From left to right, EtOH:water ratio: 1:9, 2:8. 3:7, 4:6, 5:5, 6:4, 7:3, 8:2, 9:1 (v/v). Incubation time of 5 min.


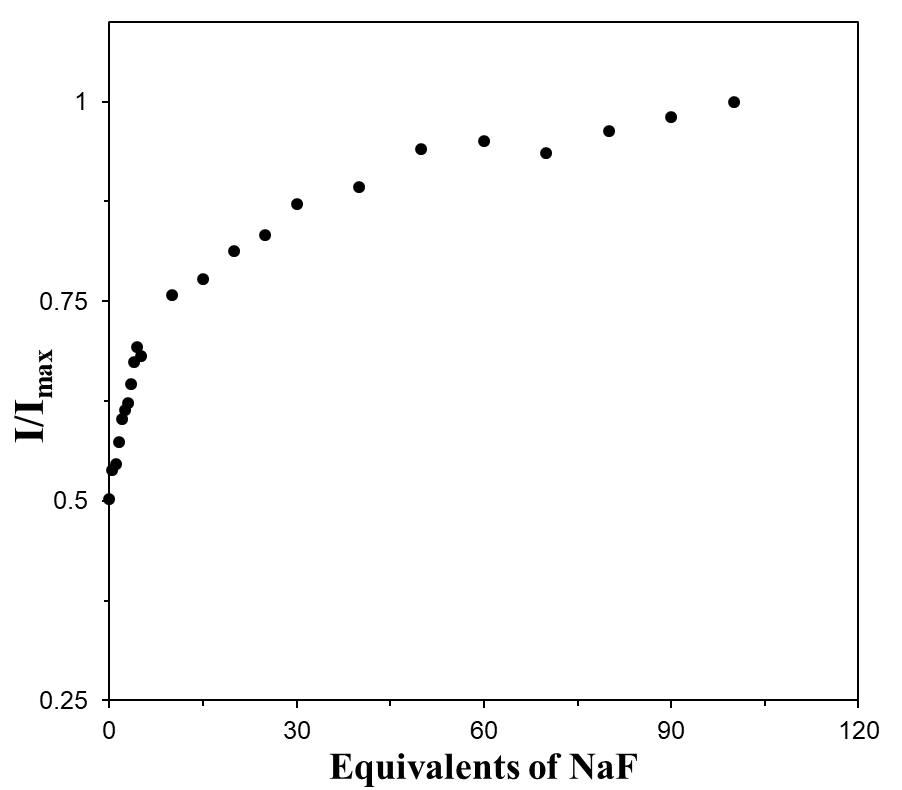


**Figure S8**. UV-vis absorbance dose dependence curve (λ_max_ = 462 nm) of NAz-6-Bpin (0.5 mM) and NaF in EtOH:H_2_O, 3:7, v/v.

**Figure S9**. Langmuir Isotherm plot of the normalised intensity of absorption of NAz-6-Bpin (0.5 mM) at 462 nm against concentration of NaF, plotted using **Equation 1**. A K_D_ of 4.67 (± 0.39) mM was obtained, equating to a K_A_ of 214 M^-1^ by using **Equation 2**.

The association constant (K_A_) of NAz-6-Bpin in EtOH:H_2_O (3:7) with NaF was calculated using the following equations:

| $y=\frac{{(I-I_{0})}_{max}[F]}{K_{D}+[F]}$ | **(1)** |
| --- | --- |
| $K_{A}=\frac{1}{K_{D}}$ | **(2)** |

Where (I-I_0_)_max_ is 0.293 (±0.007), [F] is the concentration of fluoride, K_D_ is dissociation constant and K_A_ is the association constant.


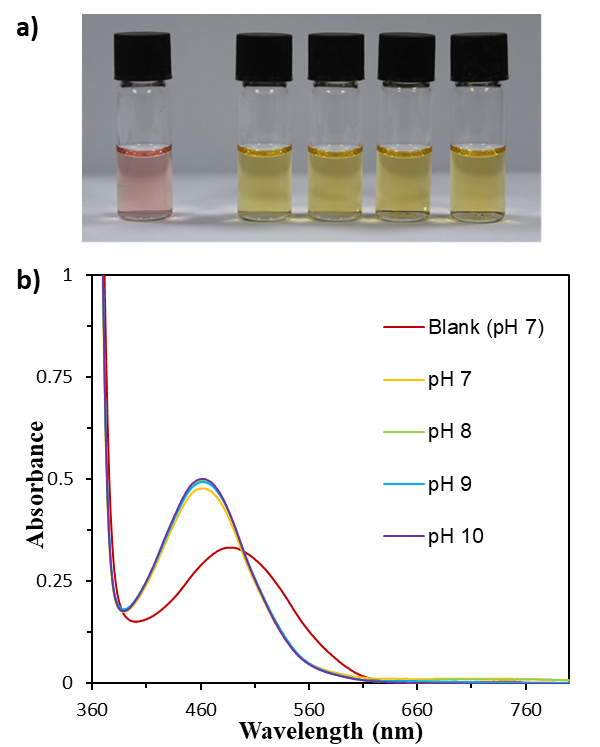


**Figure S10**. Assessment of NAz-6-Bpin (0.5 mM) for detection of NaF (60 eqv) in EtOH:H_2_O, 3:7, v/v at basic pHs. (a) From left to right: blank at pH 7, pH 7, pH 8, pH 9, pH 10. (b) UV-vis spectra of the samples pictured. Incubation time of 5 min.

**
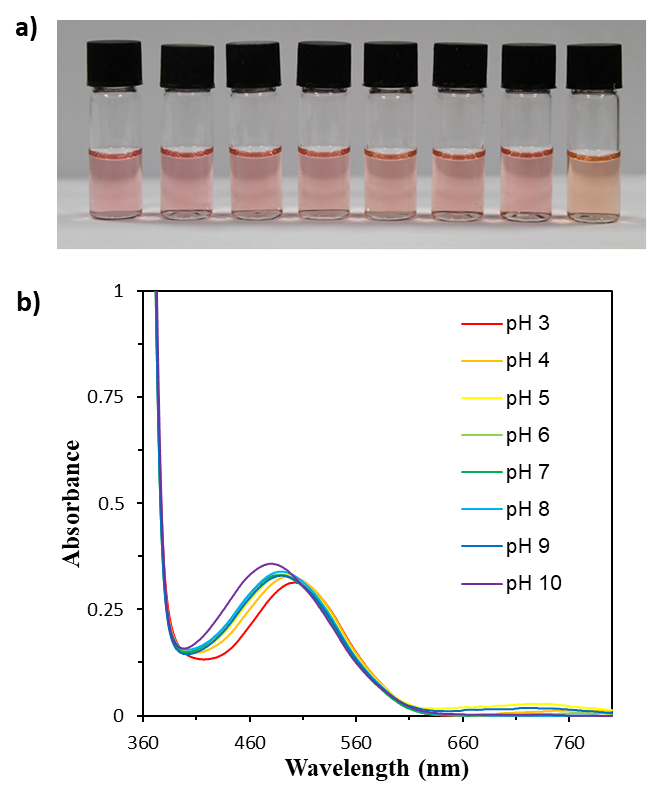
**

**Figure S11**. Stability of NAz-6-Bpin (0.5 mM) in EtOH:H_2_O, 3:7, v/v at various pHs. (a) From left to right: blank at pH 3 – 10. (b) UV-vis spectra of the samples pictured. Incubation time of 5 min.


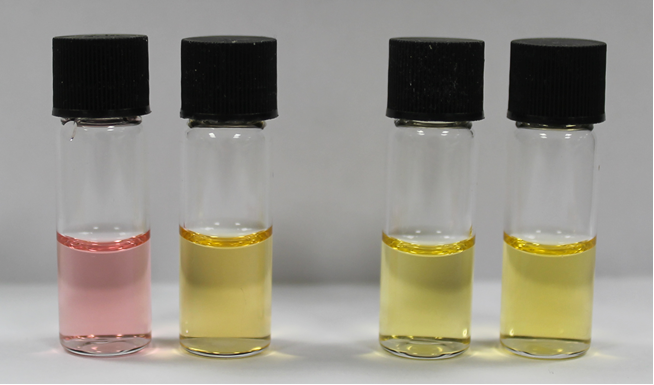


**Figure S12**. Comparison of NAz-6-Bpin (0.5 mM) in either a EtOH:water, 3:7, v/v system (*left*) or a EtOH:PBS, 3:7, v/v system (*right*). From left to right: blank (water), 60 eqv NaF (water), blank (PBS), 60 eqv NaF (PBS). Incubation time of 5 min.


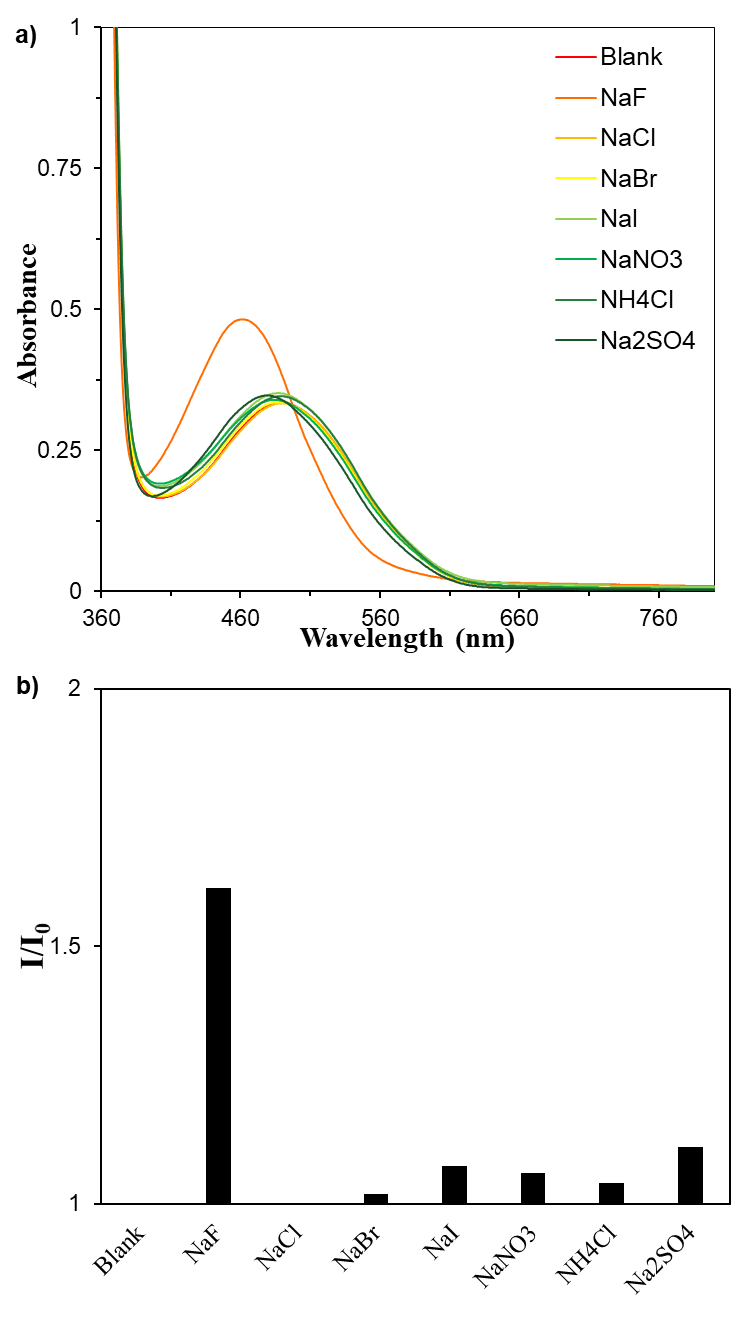


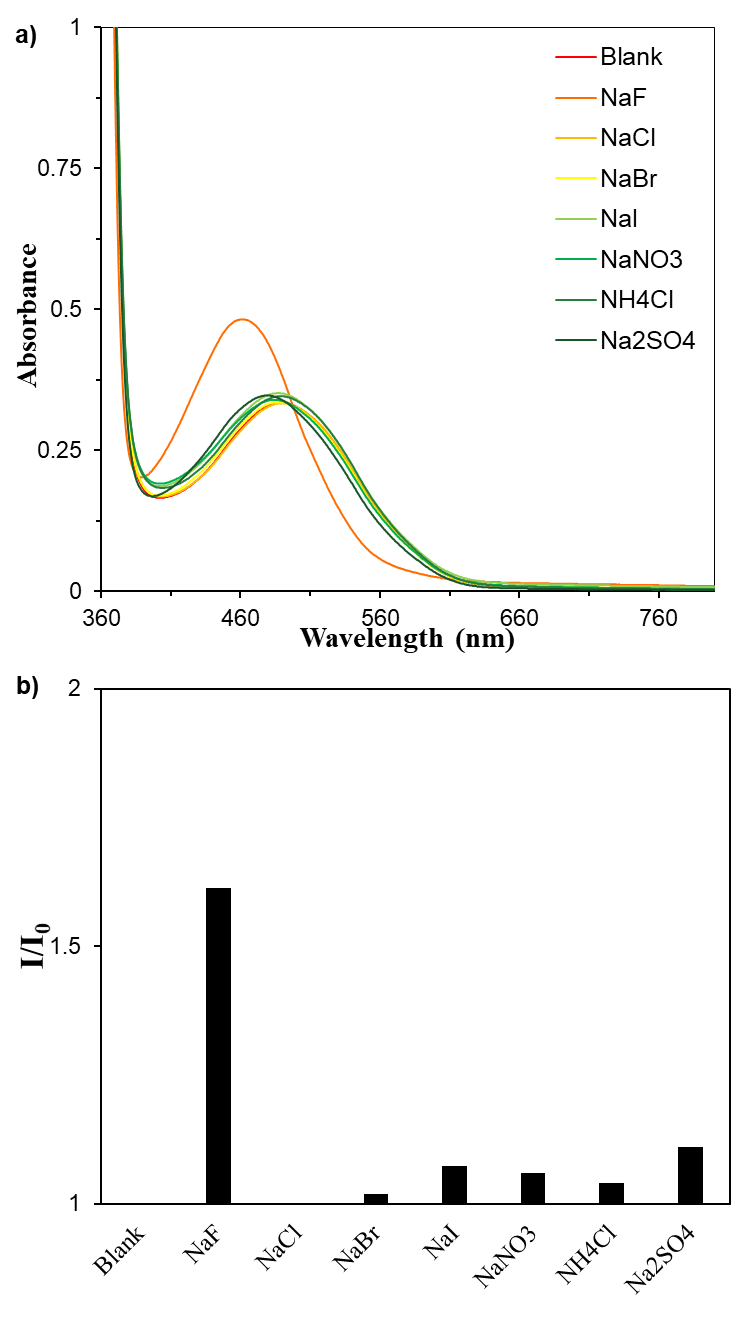


**Figure S13**. a) UV-vis selectivity test of NAz-6-Bpin in EtOH:H_2_O, 3:7, v/v (0.5 mM), with 60 equivalents of analyte used. b) Comparison of absorbance intensity of NAz-6-Bpin and analytes at 462 nm.


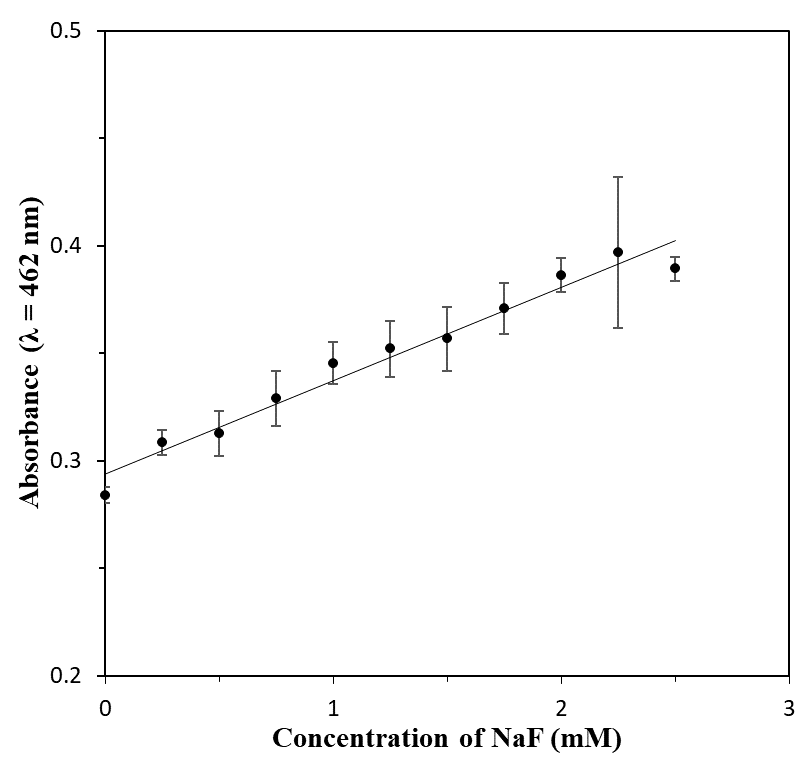


**Figure S14**. UV-vis limit of detection of NAz-6-Bpin in EtOH:H_2_O, 3:7, v/v (0.5 mM) determined by UV-vis absorption at 462 nm with NaF, calculated to be 5.75 mg L^-1^. Displayed errors calculated by standard deviation, n =3.

## Limit Of Detection Calculation

Limit of detection (LOD) was calculated using **Equation 3**.

| $LOD= \frac{3.3\sigma}{m}$ | **(3)** |
| --- | --- |

Where *σ* is the standard deviation of the blank and *m* is the gradient of the line of best fit.

**
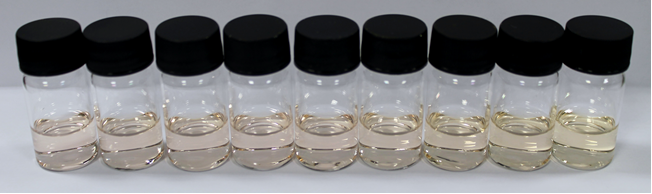
**

**
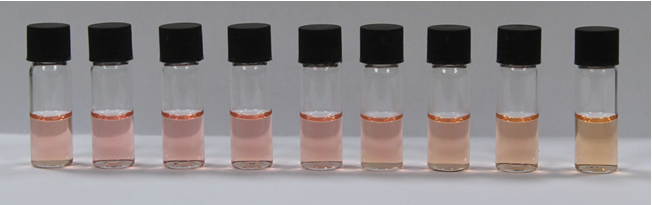
**

**Figure S15**. Visual limit of detection of NAz-6-Bpin in EtOH:H_2_O, 3:7, v/v. Top: low sensor concentration (0.5 mM). Bottom: high sensor concentration (0.1 mM). In both images, each vial contains (from left to right): blank, 2, 4, 6, 8, 10, 20, 40, 60 mg L^-1^ of NaF. Incubation time of 5 min.

**References**

Holovics, T. C., Robinson, R. E., Weintrob, E. C., Toriyama, M., Lushington, G. H., Barybin, M. V. (2006). The 2,6-diisocyanoazulene motif: Synthesis and efficient mono- and heterobimetallic complexation with controlled orientation of the azulenic dipole. *J. Am. Chem. Soc.* 128, 2300-2309. doi:10.1021/ja053933+

Kurotobi, K., Tabata, H., Miyauchi, M., Murafuji, T., Sugihara, Y. (2002). Coupling reaction of azulenyl-4,4,5,5-tetramethyl-1,3,2-dioxaborolanes with haloazulenes. *Synthesis-Stuttgart* 8, 1013-1016. doi:10.1055/s-2002-31947

Nolting, D. D., Nickels, M, Price, R., Gore, J. C., Pham, W. (2009). Synthesis of bicyclo 5.3.0 azulene derivatives. *Nat. Protoc.* 4, 1113-1117. doi:10.1038/nprot.2009.99

Nozoe, T., Takase, K., Kato, M., Nogi, T. (1971). Reaction of 2-arylsulfonyloxytropones and active methylene compounds. The formation of 8-hydroxy-2H-cyclohepta[b]furan-2-one and 2-amino-8H-cyclohepta[b]furan-8-one derivatives. *Tetrahedron* 27, 6023-6035. doi:10.1016/s0040-4020(01)91767-3

Yang, C., Schellhammer, K. S., Ortmann, F., Sun, S., Dong, R., Karakus, M., Mics, Z., Loeffler, M., Zhang, F., Zhuang, X., Canovas, E., Cuniberti, G., Bonn, M., Feng, X. (2017). Coordination Polymer Framework Based On-Chip Micro-Supercapacitors with AC Line-Filtering Performance. *Angew. Chem. Int. Ed.* 56, 3920-3924. doi:10.1002/anie.201700679
